# Persistent inflammation does not promote aversion-resistant binge-like alcohol drinking in rats

**DOI:** 10.1016/j.alcohol.2025.07.003

**Published:** 2025-07-29

**Authors:** Jessica A. Cucinello-Ragland, Yolanda Campos-Jurado, Lila Hershfelt, Mateo Pujol, Youssef Saad, Bilal Zahoor, Alexandre Neptune, Jose A. Morón

**Affiliations:** a Department of Anesthesiology, Washington University in St. Louis, St. Louis, MO, USA; b Pain Center, Washington University in St. Louis, St. Louis, MO, USA; c School of Medicine, Washington University in St. Louis, St. Louis, MO, USA; d Department of Neuroscience, Washington University in St. Louis, St. Louis, MO, USA; e Department of Psychiatry, Washington University in St. Louis, St. Louis, MO, USA

**Keywords:** Pain, Quinine, Alcohol

## Abstract

**Background:**

Chronic pain is a leading cause of disability, significantly decreases quality of life, and is highly comorbid with substance use disorders, including alcohol use disorder (AUD). This is due, in part, to the pain-relieving effects of alcohol acting as a potential driving force for the progression and maintenance of AUD. Despite a substantial body of historic, anecdotal, clinical, and epidemiological evidence supporting the analgesic efficacy of alcohol, few preclinical studies have investigated the effects of pain on volitional alcohol drinking. Further, no studies to date have investigated aversion-resistant drinking in the context of persistent pain.

**Methods:**

To address this gap in the literature, the current study combined quinine adulteration with the drinking in the dark (DID) model of binge-like alcohol drinking to assess the effects of complete Freund’s adjuvant (CFA)-induced persistent inflammation on aversion-resistant binge-like alcohol drinking in female and male Long Evans rats.

**Results:**

Consistent with previous findings from our laboratory, CFA did not affect binge-like alcohol drinking in either sex, although female rats did consume greater levels of alcohol during baseline and post-CFA DID sessions. Similarly, CFA did not affect quinine adulterated binge-like alcohol drinking in either sex.

**Conclusions:**

This study is the first to investigate the impact of persistent inflammation on aversion-resistant alcohol drinking. Although we found no effects of CFA on quinine adulterated binge-like alcohol drinking, these findings provide the groundwork for future investigations into this otherwise unstudied aspect of the pain-alcohol relationship.

## 1. Introduction

Chronic pain is a growing and significant public health concern, affects approximately 10–20 % of the global population (Andrews et al., 2018; Zimmer et al., 2022) and 20 % of adults in the United States (Yong et al., 2022), and is a leading cause of disability (GBD 2016 Disease and Injury Incidence and Prevalence Collaborators, 2017). Further, pain can also act as a driver for the onset of alcohol drinking and development and maintenance of alcohol use disorder (AUD), in part due to the pain-relieving, or analgesic properties of acute alcohol (Edwards et al., 2020; Egli et al., 2012). Although the analgesic effects of alcohol have been known for millennia (Crocq, 2007; McGovern et al., 2009), few preclinical studies have investigated the effects of pain on alcohol drinking (Cucinello-Ragland & Edwards, 2021), and the pain and alcohol fields rarely consider the others’ impact (Witkiewitz & Vowles, 2018). However, clinical and epidemiological studies commonly report pain-driven alcohol use. For example, alcohol drinking for pain management is reported in 25 % of chronic pain patients (Riley & King, 2009) and 79 % of high-risk alcohol drinkers (Alford et al., 2016), and the presence of pain as well as pain intensity are associated with an increased urge to drink alcohol (Moskal et al., 2018), increased alcohol consumption (Von Korff et al., 2005; Witkiewitz et al., 2015a), and increased rates of hazardous drinking (Ngo et al., 2021; Paulus et al., 2020; Witkiewitz et al., 2015b).

The effects of persistent pain on volitional alcohol drinking in preclinical models are varied, in part due to variability in pain model used, onset of pain in relation to alcohol drinking, and drinking model used (Campos-Jurado & Morón, 2023). Given this, different pain models have been shown to either decrease, increase, or have no effect on alcohol consumption. Despite clinical evidence indicating that alcohol-induced analgesia occurs at or above a blood alcohol content of .08 % (Thompson et al., 2017) and preclinical evidence that binge-like doses of alcohol attenuate complete Freund’s adjuvant (CFA)-induced hypersensitivity (Cucinello-Ragland et al., 2023), few preclinical studies have investigated the effects of persistent pain on binge-like alcohol drinking.

Prior work from our laboratory found that CFA-induced persistent inflammatory pain blunts concentration-dependent decreased alcohol intake only in male rats, while there is no effect of CFA treatment on alcohol consumption at any concentration in female rats (Campos-Jurado & Morón, 2023). Given the relative aversiveness of these high concentrations of alcohol (up to 50 %) used in our previous study, the present work sought to determine if CFA-induced blunting of decreased alcohol consumption at high concentrations was due to compulsive-like or aversion-resistant alcohol drinking. Further, little work has been done investigating how persistent pain relates to compulsive-like alcohol drinking. To address these deficits in preclinical studies, the present study examines binge-like and quinine adulterated alcohol consumption in female and male Long Evans rats injected in the hind paw with CFA to model persistent inflammatory pain. The CFA model was chosen given its relevant time course of persistent injury (Hargreaves et al., 1988; Larson et al., 1986; Ren & Dubner, 1999), extended persistence of hypersensitivity (Burek et al., 2022), and the high prevalence of inflammatory pain complaints. Our results show that persistent inflammation does not affect either binge-like alcohol drinking or quinine adulterated drinking in either sex, suggesting that our previous findings reporting a pain-induced shift in the dose-response to alcohol are not due to aversion-resistant drinking.

## 2. Materials and methods

### 2.1. Animals

Adult (8–12 weeks old at start of experiment) female and male Long Evans rats (N = 39; bred in-house, breeders purchased from Envigo) were single housed and given *ad libitum* access to food and water throughout the duration of the experiment. Rats were maintained on a reverse 12-h light/dark cycle, handled regularly, and allowed one week to acclimate to single-housing, reverse light cycling, and two water bottles in the cage prior to the start of experimental procedures. All animal care, use, and procedures in this study were approved by the Institutional Animal Care and Use Committee of Washington University in St. Louis and were in accordance with the National Institute of Health guidelines.

### 2.2. Drinking in the dark model of binge-like drinking

Binge-like alcohol drinking was modeled using a two-bottle choice drinking in the dark (DID) paradigm. Rats were allowed one week to acclimate to two water bottles in the home cage prior to the start of experimental procedures. 3 to 4 h after the start of the dark phase of the light/dark cycle, bottles were weighed, and one of the two water bottles was replaced with a bottle containing 20 % v/v ethanol (side switched each session). Animals were allowed to drink *ad libitum* from both bottles for 4 h, after which time the ethanol and water bottles were weighed and the ethanol bottle was replaced with water. The difference in the weight of the bottles at the start and end of the session were used to calculate the volume of liquid consumed. Bottles were fitted with double ball bearing sippers (5/16″ diameter, 1.5” length; Ancare, cat# TD-99) to minimize leakage. DID sessions occurred Monday-Friday, and two bottles of water remained in the cage at all times when DID sessions were not occurring.

### 2.3. Quinine adulteration model of compulsive-like drinking

Given our previous work identifying sex-dependent effects of persistent inflammatory pain on alcohol consumption at high concentrations, we chose to utilize the quinine adulteration model of compulsive-like-drinking. In the final week of the experiment, all animals underwent a quinine challenge during the DID procedure in which they received a bottle containing alcohol mixed with the bitter tastant quinine hydrochloride (Sigma-Aldrich, cat# Q1125) at escalating concentrations (0, 0.1, 0.3, 0.5, 1.0 g/L). The difference in the weight of the bottles at the start and end of the session were used to calculate the volume of liquid consumed.

### 2.4. Complete Freund’s adjuvant model of persistent inflammatory pain

Persistent inflammation was modeled using the complete Freund’s adjuvant (CFA) model as previously described (Campos-Jurado & Morón, 2023; Cucinello-Ragland et al., 2023). Briefly, rats were anesthetized with 5 % isoflurane prior to subcutaneous intraplantar hind paw injection with 120uL (females) or 150uL (males) of either 0.9 % sterile saline (Control) or 50 % CFA in saline (ThermoFisher, cat# 77140) into the left or right hind paw (balanced across groups) using a 26-gauge needle. Group assignments were counterbalanced by sex based on baseline ethanol intake (g/kg) and alcohol preference. Hind paw injections occurred 3 days before the start of Week 3 DID based on previous work from our laboratory (Higginbotham et al., 2025). Hind paw edema was visually confirmed following hind paw injection during routine wellness checks prior to the first post-CFA drinking session to confirm that CFA-induced edema was localized to the hind paw and did not spread up the limb and that saline injection did not produce edema or visual tissue injury. Although this was not observed in any animal in the present study, we regularly confirm localization of CFA-induced edema to ensure animal welfare, and any animal exhibiting spreading of edema would have been immediately excluded from the study and euthanized.

### 2.5. Statistics

All data were analyzed using Prism 10 (GraphPad Software, Inc., La Jolla, CA, USA). Data were analyzed using repeated measured three-way ANOVA (CFA x Sex x Time; CFA x Sex x Quinine Concentration). Statistical significance was set as *p* < 0.05. Data collection and analysis were performed blinded to the conditions of the experiments. All data are expressed as mean ± SEM.

## 3. Results

### 3.1. CFA does not alter binge-like alcohol drinking

**Fig. 1.**
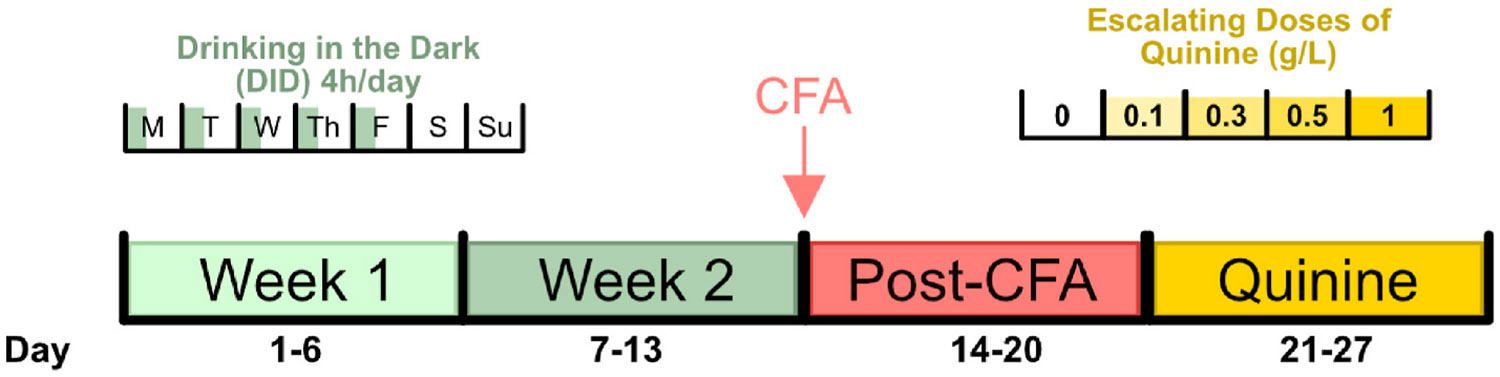
Experimental timeline.

**Fig. 2.**
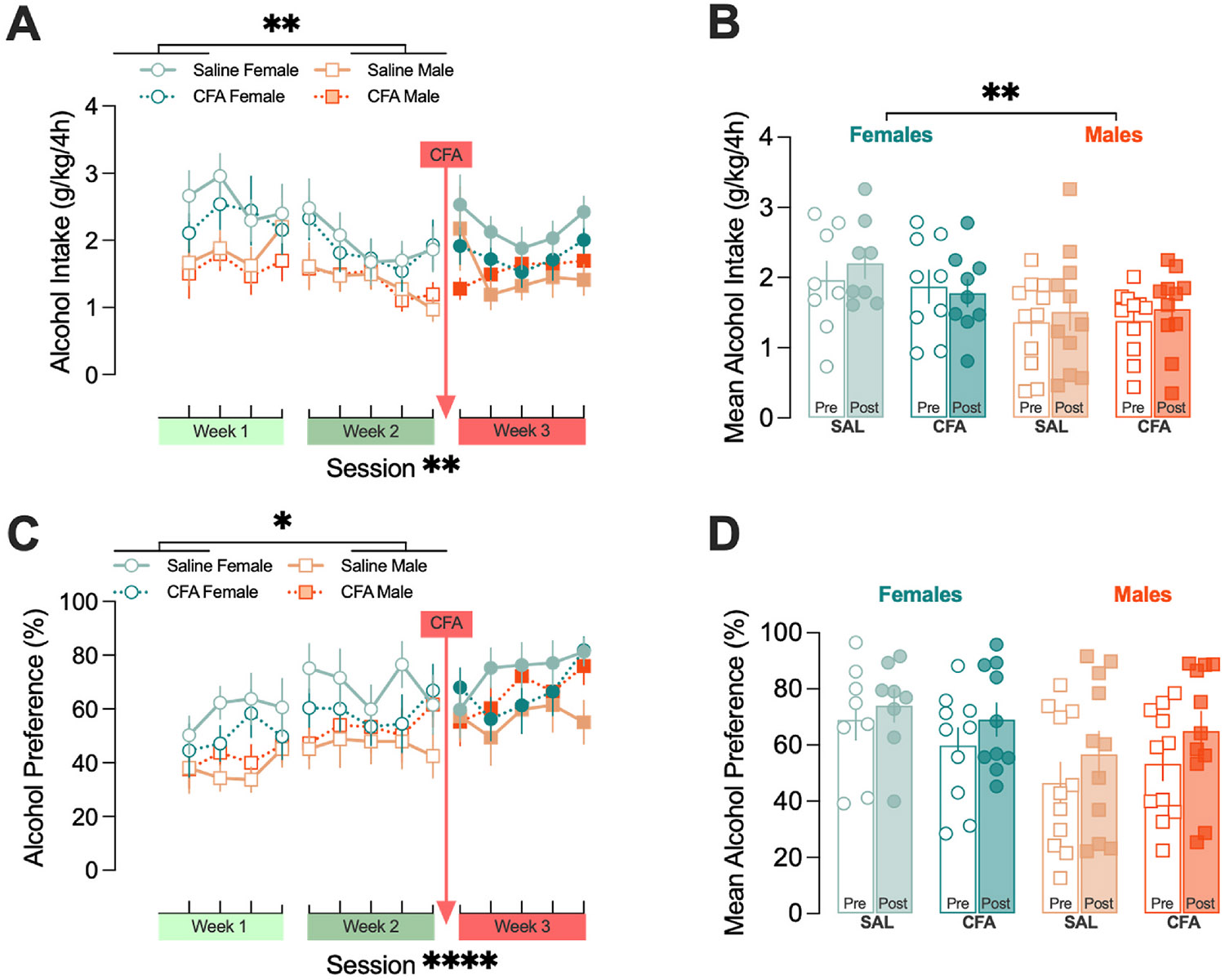
Persistent inflammation does not affect binge-like alcohol drinking. Data are shown by alcohol intake (grams of alcohol consumed per kilograms of body weight, A-B) and alcohol preference (percent of alcohol consumed over total fluid intake, C-D). Females consumed more alcohol (A; *p* = 0.0021) and had higher alcohol preference (C; *p* = 0.0242) than males across all pre-quinine sessions. This was also true when comparing the average of the 5 sessions prior to CFA injection and the 5 sessions following CFA injection (B, *p* = 0.0084; D, *p* = 0.0544). There was also a main effect of time to increase alcohol intake (A; *p* = 0.0012) and alcohol preference (C; *p* < 0.0001). Data were analyzed using repeated measures three-way ANOVA and are represented as mean ± SEM. Females are represented in circular symbols in shades of teal (dark teal: saline controls, n = 8; light teal: CFA, n = 9). Males are represented in square symbols in shades of orange (dark orange: saline controls, n = 11; light teal: CFA, n = 11). Open symbols and bars indicate pre-CFA sessions, closed symbols and bars indicate post-CFA sessions. Data are included for every animal during every session except for one saline female and one CFA male on session 3 due to flipping one or both of their bottles upward out of the grate during the session. Main effects are indicated by * *p* < 0.05, **p < 0.01, and *****p < 0.0001.

Because most people with chronic pain have some history of alcohol use prior to pain onset, and given rats’ natural propensity against alcohol drinking, animals were exposed to 9 drinking sessions over the course of two weeks to establish a stable baseline level of alcohol consumption prior to hind-paw injection. Rats were then given 5 additional DID sessions to assess the effects of CFA treatment on binge-like alcohol drinking (experimental timeline outlined in Fig. 1). Consistent with our previous findings using the intermittent access two bottle choice paradigm (Campos-Jurado & Morón, 2023), CFA treatment did not affect alcohol intake (grams of alcohol consumed per kilograms of body weight; Fig. 2A: CFA: F_1,35_ = 0.8077, *p* = 0.3750, CFA x sex interaction: F_1,35_ = 0.4332, *p* = 0.5147; Fig. 2B: CFA: F_1,35_ = 0.4056, *p* = 0.5283, CFA x sex interaction: F_1,35_ = 0.6428, *p* = 0.4281) or alcohol preference (percent of alcohol consumed over total fluid intake; Fig. 2C: CFA: F_1,35_ = 0.0310, *p* = 0.8612, CFA x sex interaction: F_1,35_ = 2.198, *p* = 0.1471; Fig. 2D: CFA: F_1,36_ = 0.0017, *p* = 0.9670, CFA x sex interaction: F_1,36_ = 1.331, *p* = 0.2562) in either sex in the DID model. There were additionally no significant CFA interactions on either alcohol intake (Fig. 2A: time x CFA: F_13,452_ = 0.7217, *p* = 0.7422, time x CFA x sex: F_13,452_ = 0.4487, *p* = 0.9509; Fig. 2B: time x CFA: F_1,35_ = 0.4468, *p* = 0.5082, time x CFA x sex: F_1,35_ = 0.6149, *p* = 0.4382) or alcohol preference (Fig. 2C: time x CFA: F_13,452_ = 0.7565, *p* = 0.7064, time x CFA x sex: F_13,452_ = 0.5562, *p* = 0.8882; Fig. 2D: time x CFA: F_1,36_ = 0.2729, *p* = 0.6046, time x CFA x sex: F_1,36_ = 0.0601, *p* = 0.8076). There is, however, a main effect of time and sex on both alcohol intake (time: F_6.193,215.3_ = 3.765, *p* = 0.0012; sex: F_1,35_ = 10.99, *p* = 0.0021) and alcohol preference (time: F_6.280,218.4_ = 6.158, *p* < 0.0001; sex: F_1,35_ = 5.549, *p* = 0.0242) for drinking across all pre-quinine sessions (including both pre- and post- CFA sessions; Fig. 2A and C).

### 3.2. CFA does not alter quinine-adulterated drinking

**Fig. 3.**
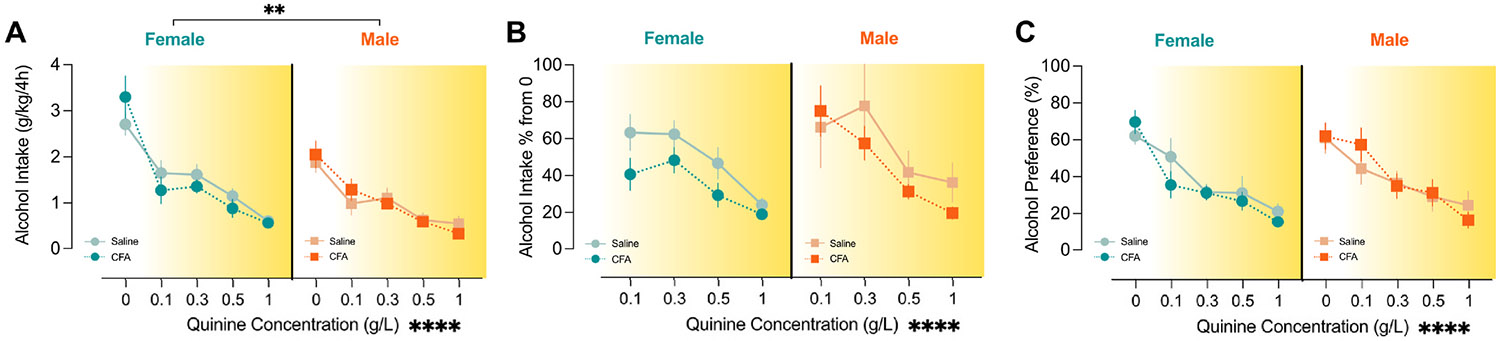
Persistent inflammation does not affect quinine adulterated binge-like alcohol drinking. Data are shown by alcohol intake (grams of alcohol consumed per kilograms of body weight, A), alcohol intake relative to alcohol intake consumed during the 0 g/L session (B), and alcohol preference (percent of alcohol consumed over total fluid intake, C). Across all analyses, there was a main effect of quinine concentration to decrease alcohol intake and preference (*p* < 0.0001 for all). There was a main effect of sex on total alcohol intake (A; *p* = 0.0021). Data were analyzed using repeated measures three-way ANOVA and are represented as mean ± SEM. Females are represented in circular symbols in shades of teal (dark teal: saline controls, n = 8; light teal: CFA, n = 9). Males are represented in square symbols in shades of orange (dark orange: saline controls, n = 11; light teal: CFA, n = 11). Data are included for every animal during every session except for one saline female and one saline male on the 0 g/L session due to flipping one or both of their bottles upward out of the grate during the session. Main effects are indicated by **p < 0.01 and *****p < 0.0001.

Following a week of post-CFA drinking sessions, the alcohol-containing water bottle was adulterated with quinine at escalating doses presented in different sessions. As expected, there was a main effect of quinine concentration to decrease overall alcohol intake (Fig. 3A; F_1.918,67.13_ = 67.84, *p* < 0.0001), alcohol intake relative to the 0 g/L quinine concentration given on the quinine challenge week (Fig. 3B; F_1.918,67.13_ = 67.84, *p* < 0.0001 and alcohol preference (Fig. 3C; F_3.346,115.4_ = 40.27, *p* < 0.0001). There was a main effect of sex (F_1,35_ = 11.09, *p* = 0.0021) and a quinine concentration x sex interaction (F_4,140_ = 3.395, *p* = 0.0110) on alcohol intake during quinine challenge (Fig. 3A). However, given that females consumed greater levels of alcohol prior to quinine adulteration (Fig. 2), we analyzed alcohol intake relative to the 0 g/L quinine concentration given on the quinine challenge week (Fig. 3B). In this analysis, there was no significant effect of sex (main effect of sex: F_1,35_ = 0.7146, *p* = 0.4047), suggesting that the sex effect observed in overall quinine-adulterated alcohol intake is driven by greater levels of unadulterated alcohol intake in females. Further, there was no effect of sex or a quinine concentration x sex interaction on alcohol intake relative to 0 g/L quinine (quinine concentration x sex: F_3,105_ = 1.669, *p* = 0.1782) or alcohol preference (main effect of sex: F_1,35_ = 0.1844, *p* = 0.6703; quinine concentration x sex: F_4,138_ = 0.6759, *p* = 0.6098). There were no effects of CFA treatment or interactions between CFA and any other variable on overall alcohol intake (main effect of CFA: F_1,35_ = 0.03396, *p* = 0.8549; CFA x quinine concentration: F_4,140_ = 1.676, *p* = 0.1589; CFA x sex: F_1,35_ = 0.09545, *p* = 0.7592; CFA x sex x quinine concentration: F_4,140_ = 1.305, *p* = 0.2713), alcohol intake relative to unadulterated alcohol consumption on the quinine challenge week (main effect of CFA: F_1,35_ = 1.33, *p* = 0.2566; CFA x quinine concentration: F_3,105_ = 0.4362, *p* = 0.7275; CFA x sex: F_1,35_ = 0.06050, *p* = 0.8071; CFA x sex x quinine concentration: F_3,105_ = 2.083, *p* = 0.1069), or alcohol preference (main effect of CFA: F_1,35_ = 0.04295, *p* = 0.8370; CFA x quinine concentration: F_4,138_ = 0.6241, *p* = 0.6461; CFA x sex: F_1,35_ = 0.2186, *p* = 0.6430; CFA x sex x quinine concentration: F_4,138_ = 1.735, *p* = 0.1458).

## 4. Discussion

Based on the bidirectional relationship between pain and alcohol consumption (Egli et al., 2012; Robins et al., 2019; Zale et al., 2015), as well as the use of alcohol for pain relief in people with chronic pain (Alford et al., 2016; Brennan et al., 2005; Karimi et al., 2022; Stein et al., 2024), this study investigated the effects of persistent inflammation on binge-like and quinine adulterated alcohol drinking. While much preclinical work has been done characterizing the development of hyperalgesia and increased pain reactivity in animal models of AUD (Brandner et al., 2023; Dina et al., 2008; Kelley et al., 2024; Edwards et al., 2012; Roltsch Hellard et al., 2017; Secci et al., 2024), fewer studies have investigated the role of pain models on volitional alcohol drinking (Campos-Jurado & Morón, 2023; Cucinello-Ragland & Edwards, 2021; Cuitavi et al., 2025; Lorente et al., 2022). Within this, even fewer have focused on binge-like alcohol drinking, despite meta-analysis evidence that analgesia is achieved at binge levels of alcohol consumption (Thompson et al., 2017). Further, the effects of pain on compulsive-like alcohol drinking, or drinking despite negative consequences, have been relatively unstudied in both the preclinical and clinical settings.

In the current study, we first determined the effects of persistent inflammation on binge-like alcohol drinking in rats with a history of alcohol drinking. Given the effects of inflammatory signaling and negative affective state on alcohol drinking and reward, our initial hypothesis was that CFA treatment would increase binge-like alcohol drinking (Blednov et al., 2011; Tovmasyan et al., 2022; Vetreno & Crews, 2014; Zale et al., 2015). Further, despite our previous findings that CFA does not alter consumption of 20 % v/v alcohol (Campos-Jurado & Morón, 2023), we anticipated CFA-induced increases in drinking under a DID schedule based on existing literature that binge levels of alcohol are highly analgesic (Cucinello-Ragland et al., 2023; Thompson et al., 2017). Our findings indicate that the CFA model of persistent inflammation does not increase home cage binge-like alcohol drinking in the DID model in either female or male Long Evans rats. Although one study of sciatic nerve injury in mice reported increased DID alcohol consumption in the pain condition (González-Sepúlveda et al., 2016), our findings are consistent with studies reporting no effects of CFA treatment on intermittent and continuous access two bottle choice drinking (Cuitavi et al., 2025; McGinn et al., 2020; Smith et al., 2015). Further, the present study also found that females consumed more alcohol than males regardless of pain status, consistent with previous reports of home cage drinking in rats (Lancaster & Spiegel, 1992; Morales et al., 2015; Priddy et al., 2017) and mice (Satta et al., 2018; Sneddon et al., 2019). It is important to note, however, that the levels of alcohol consumption observed in the present study may be at the upper limit of possible alcohol consumption in the two-bottle choice DID model. While less reported in the literature in rats than mice, Holgate and colleagues reported an average alcohol consumption in the two-bottle choice DID model in male Wistar rats as observed in the present study across sexes (Holgate et al., 2017). Although the DID model was chosen based on its translational relevance, the short duration of drinking sessions compared to other home cage drinking paradigms may preclude our ability to observe increased intake following CFA treatment.

To our knowledge, the present study is the first of its kind investigating compulsive-like alcohol drinking in the context of a persistent pain model. Although we hypothesized that CFA treatment would promote aversion-resistant drinking in male rats based on our previous findings, we found no effects of persistent inflammation on quinine adulterated drinking. While our initial analysis of adulterated alcohol intake indicated a potential sex difference, further analyses indicated that this main effect was primarily driven by greater levels of alcohol consumption in females in the absence of quinine. This lack of sex differences in quinine-adulterated alcohol consumption are consistent with mixed reports in the literature. Indeed, sex differences in aversion-resistant alcohol drinking vary across experiments, drinking model, and species (Maddern et al., 2024). Although Sneddon and colleagues found that female mice consume more quinine-adulterated alcohol under DID conditions (Sneddon et al., 2019), a similar study reported no sex differences in quinine-adulterated alcohol consumption in mice following 3 weeks of DID (Bauer et al., 2021). Further, there are mixed reports on the effects of sex on quinine-adulterated alcohol consumption across increasing concentrations of quinine. While female and male mice drinking in a limited-access two-bottle choice paradigm display similar levels of alcohol drinking across increasing concentrations of quinine (Sneddon et al., 2019), under continuous access conditions, female mice require higher concentrations of quinine to suppress alcohol drinking (Fulenwider et al., 2019). Although female rats display greater levels of aversion-resistant drinking in home cage (Radke et al., 2020) and operant (Randall et al., 2017) alcohol drinking, these studies exposed animals to much longer durations of alcohol consumption (home cage: 5–6 and 11–12 weeks of intermittent access two-bottle choice; operant: 12 weeks of self-administration followed by 3 weeks of abstinence and 2 weeks of extinction) prior to quinine challenge than the present study. Given this, our lack of observed sex differences in quinine-adulterated alcohol intake may be precluded by the relatively short duration of alcohol drinking history prior to quinine challenge.

Despite our lack of CFA effects on quinine adulterated drinking, there are several factors to consider for future investigations into the role of pain in compulsive-like alcohol drinking. First, the present study utilized animals with a brief history of alcohol drinking to better model the clinical population. However, this history of alcohol consumption may drive increased alcohol consumption and preclude potential effects of CFA treatment. Further, although CFA was chosen due to its translational relevance, more severe pain models, such as those modeling neuropathic pain, may differentially effect aversion-resistant drinking. While findings of escalation of alcohol consumption following inflammatory pain are mixed (Campos-Jurado & Morón, 2023), there are more consistent reports of neuropathic pain-facilitated alcohol consumption. Greater levels of alcohol consumption are observed in mice following sciatic nerve ligation and spared nerve injury (Bilbao et al., 2019; González-Sepúlveda et al., 2016). Further, analgesic efficacy is diminished over time in mice with neuropathic, but not inflammatory, pain, providing a potential cause for escalated alcohol consumption under neuropathic pain conditions (Neddenriep et al., 2019). Importantly, neither inflammatory or neuropathic pain models fully capture chronic pain as observed clinically, which is normally a complex, heterogenous combination of both inflammatory and neuropathic mechanisms. As a further limitation, while the CFA model of persistent inflammatory pain is well-established and validated in both our laboratory and the field as a whole, the present study did not include assessment of nociceptive testing due to technical constraints that would have introduced additional, unnecessary stressors to the animals. Our previous work shows that the CFA model produces similar hypersensitivity in both female and male rats, measured as far out as 40 days post-hind paw injection (Campos-Jurado & Morón, 2023; Cucinello-Ragland et al., 2023; Higginbotham et al., 2025). While pain phenotyping was not possible in the present study, based on historical validation of this model (Burek et al., 2022; Fehrenbacher et al., 2012; Ferdousi et al., 2023; Ren & Dubner, 1999; Wilson et al., 2006), we are confident that inflammatory pain was present in these animals. Although McGinn and colleagues found no effect of CFA on home-cage alcohol drinking in male rats, they reported temporal changes in the relationships between cold nociceptive sensitivity and alcohol drinking throughout the chronification of CFA (McGinn et al., 2020). Given this, lack of pain confirmation is an important limitation of the present studies that should be addressed in any future investigations of pain-modulated aversion-resistant alcohol drinking. Future studies addressing these constraints are necessary to fully assess the relationship between pain and compulsive-like alcohol drinking, providing new insights into understudied and poorly understood aspects of the pain-alcohol relationship.
